# Possibility of New Active Substrates (ASs) to Be Used to Prevent the Migration of Heavy Metals to the Soil and Water Environments

**DOI:** 10.3390/molecules28010094

**Published:** 2022-12-22

**Authors:** Katarzyna Witt, Waldemar Studziński, Daria Bożejewicz

**Affiliations:** Faculty of Chemical Technology and Engineering, Bydgoszcz University of Science and Technology, 3 Seminaryjna Street, PL 85326 Bydgoszcz, Poland

**Keywords:** active substrate (AS), remove of heavy metal ions, Aliquat 336, groundwater, soil

## Abstract

This paper aims to propose an alternative to the known permeable reactive barriers (PRBs). PRB is one of the methods, which is a reactive barrier placed below the ground, to clean up contaminated groundwater. New polymer active substrates (ASs) were used to prevent soil contamination by toxic heavy metals. The active substrates consisted of a mixture of poly(vinyl chloride), Aliquat 336, and bis(2-ethylhexyl)adipate, which was applied to the skeleton material (fiberglass or textile). Aliquat 336 was used as a binding agent for metal ions (Cr(VI), Ni(II), Cu(II), Zn(II), Cd(II), and Pb(II)). In contrast with the PRBs, the ASs (from **AS-1** to **AS-5**) were obtained in a simple way using the pouring method. The obtained ASs could be recycled and reused. The active substrates were used for the binding of various metal ions from aqueous solutions and the examined soil. It was found that the active substrate **AS-1** decreased the concentrations of nickel, cadmium, and lead by more than 50% and that of chromium by more than 90% in the aqueous solution. High sorption efficiency for chromium and zinc metals (81% and 66%) with the use of **AS-2** was also found, owing to which the migration of metals from soil to water can be limited. In the soil environment, active substrate **AS-5** with the addition of a plasticizer showed the greatest effectiveness. This solution resulted in a reduction in each tested metal ion of at least 50%, and reductions in cadmium, lead, and copper of over 70%.

## 1. Introduction

Heavy metals occur naturally in the environment due to the erosion of minerals, leaching metal ore deposits, and volcanic eruptions. However, the development of agriculture and industry, including the automotive industry, has caused heavy metals to penetrate environmental matrices in an anthropogenic way, e.g., because of poor waste and sewage management [1,2]. The contamination of groundwater and soil affects water and soil resources, and the presence of heavy metals in excessive concentrations may be harmful to humans and animals [3]. Therefore, heavy metal ions are the main cause of the deterioration of the quality of groundwater and soil. The condition of groundwater is deteriorating due to excessive leaching, which causes the mixing of metal ions with the groundwater layers [4]. The large amounts of toxic heavy metal ions in the natural environment (such as Cr(VI), Ni(II), Cu(II), Zn(II), Cd(II), and Pb(II)) are threats to the surface and subsurface environments. Although some heavy metals are necessary for proper growth and development in the human body, most of them are toxic to organisms, even if they are present in trace amounts. As heavy metal ions are easily accumulated in living organisms and can negatively affect life in humans and other species, it is necessary to eliminate these metal ions from contaminated water [5,6].

The reclamation of groundwater contaminated with heavy metals from natural soil sources or anthropogenic sources is a priority due to its use as drinking water. There are many technologies for the reclamation of groundwater and soil contaminated with heavy metal ions. These processes mainly aim to completely or significantly remove pollutants, extract pollutants for further purification, or remove and stabilize pollutants by transforming them into less mobile or toxic forms. Additionally, if possible, uncontaminated matrices should be separated from contaminated matrices to reduce environmental contamination and select an appropriate technology to eliminate or limit the concentrations of heavy metals [7].

**Figure 1:**
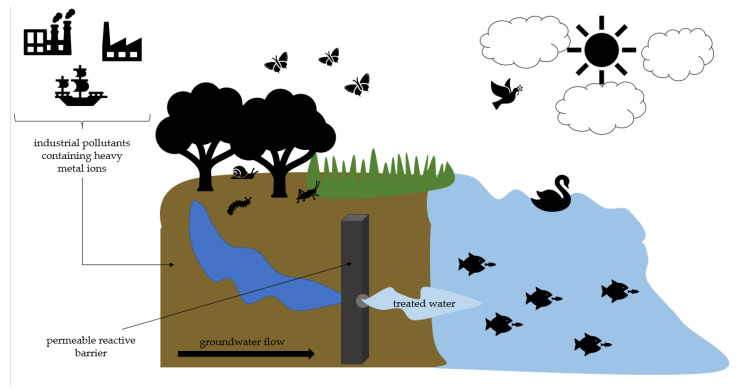
The mechanism of action of a permeable reactive barrier. Source: own graph based on Moore et al., 2016 [9].

The technologies for removing toxic metals from soil and groundwater include both permeable reactive barriers (PRBs), which consist of permeable and semi-permeable reactive materials, and the “pump and treat” active treatment technology. The traditional PRB method involves placing reactive barriers perpendicular to the potential trajectory of contaminated groundwater [8] (Figure 1).

On the other hand, the “pump and treat” method consists of pumping water out of the rock mass, subjecting it to treatment in a specially prepared purification system, and returning the clean water to the rock mass. The PRB technique has lower operating and maintenance costs than the “pump and treat” method; it includes initial costs related to the design, installation, and renovation of the area and fixed costs of monitoring the barrier’s operation. The effectiveness of PRB depends largely on the reactive factor used. For many years, the following were used as reactive factors in PRBs: zero-valent iron [10,11], waste products, and natural materials, e.g., chitin, fly ash, clay soil, zeolites [12,13], humic acids [14], polymers, and carbon materials [15,16,17,18,19]. From an economic standpoint, the main problem with PRBs is the need to remove and recover the spent reactive material after the cleaning process [20]. Therefore, recent years saw a significant growth in interest in modifying used reactive media, which may make it possible to extend the life of PRBs.

This article presents a new, yet unused, solution for binding metal ions onto the soil surface, i.e., active substrates (ASs), which prevent the penetration of these factors into groundwater. The active substrate (AS) was obtained from poly(vinyl chloride), used as a polymer matrix, Aliquat 336, used as a metal-ion-chelating reactive agent, bis(2-ethylhexyl)adipate plasticizing polymer, and a skeleton material, which was either fiberglass or textile. Ionic liquid (Aliquat 336, methyltrioctylammonium chloride) was used in the presented solution due to the strong coordination effect of the chloride anion [21]. It is commonly used in removing metal ions from aqueous solutions, e.g., in traditional solvent extraction or membrane separation processes (i.e., transport of metal ions through polymer inclusion membranes or sorption of metal ions using polymer sorption materials) [22,23,24,25,26]. Despite this, Aliquat 336 has not been used so far to bind toxic metal ions directly from the ground. The innovative nature of the conducted research, i.e., the use of Aliquat 336 in such a design as the presented active substrates (ASs), is evidenced by the fact that the authors are the first in Poland to apply for a patent for this solution (Patent Application No. P.442582). The proposed active substrates can potentially be used directly in the ground or on floors at industrial plants.

## 2. Results

### 2.1. Processes of the Sorption of Metal Ions onto Active Substrates

First, the effectiveness of metal ion sorption by the active substrate **AS-1** (Experiment 1) in an aqueous solution was tested. As previously mentioned, this experiment was aimed at assessing whether the obtained active substrates can limit migration and reduce the concentration of metal ions in water, which may, consequently, contribute to reducing the potential negative impact of heavy metals on the aquatic ecosystem.

The quantitative evaluation of the sorption process was performed using the following parameters: the metal ion sorption capacity (q_t_) of the investigated substances **AS-1**–**AS-5** and the percentage of metal ions removed from the solutions (%R_ads_), which were determined as follows:

(1) $$q_{t}=\left(\frac{c^{i}-c^{t}}{m}\right)\cdot V\;$$

(2) $$\%R_{ads}=\left(\frac{c^{i}-c^{t}}{c^{t}}\right)\cdot 100\%\;$$

where q_t_ is the sorption capacity (mg/g), V is the solution volume (L), m is the mass of the active substrate (g), and c^i^ and c^t^ are the analytical concentration of metal ions in the solution at the beginning and after a set time of sorption (mol/L), respectively [27].

The sorption capacities (q_t_) of metal ions on the investigated active substrates were calculated for the produced active substrates, which are given in Table 1.

For all substrates, except for **AS-4** and **AS-5**, the highest sorption capacities were obtained during the sorption of chromium(VI) ions. In the case of **AS-4** and **AS-5**, the highest values were obtained in the sorption processes of cadmium(II) and lead(II) ions, respectively. The lowest qt values were obtained during the sorption of zinc and copper ions for practically all tested sorbents. These results were compared with those obtained, e.g., by Martins et al. [27]. They also received lower maximum biosorption capacity for zinc (from 11.5 mg/g at 5 °C to 14.7 mg/g at 30 °C) than for cadmium (28.0 mg/g, independent of temperature). In turn, Afolabi et al. [28] reported a higher maximum adsorption capacity of banana peels for lead ions (39.32 mg/g) than for copper ions (29.26 mg/g).

#### Percentage of Metal Ion Removal from the Solutions (%R_ads_)

**Figure 2:**
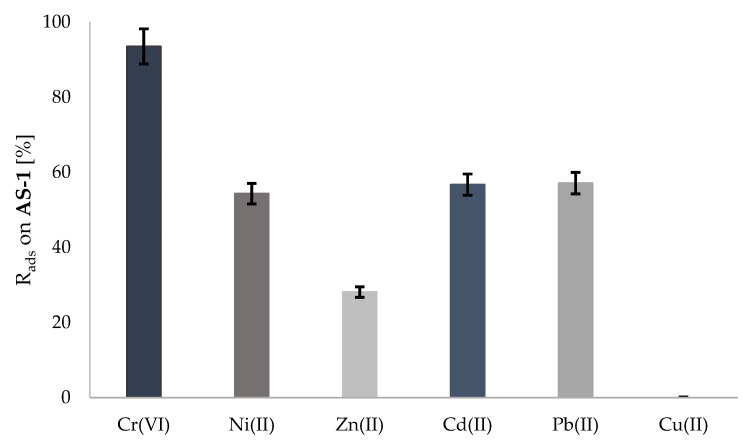
Sorption of metal ions on the active substrate (**AS-1**) according to experiment 1.

Experiment 1 was prepared in accordance with paragraph 4.5 and was carried out in order to check the effectiveness of the active substrate in an aqueous solution. The determination of metals was carried out using the AAS method, which was described in Section 4.7. Based on the conducted experiment, it was found that the active substrate **AS-1** was highly effective in removing the chromium ions from the aqueous solution. The amount of chromium(VI) ions bound with **AS-1** was 93.5%. In addition, good degrees of metal ion sorption were observed for the following metal ions: lead(II), 57.10%; cadmium(II), 56.70%; and nickel(II), 54.30%. **AS-1** exhibited the lowest effectiveness in the case of zinc (the degree of removal of this ion was 28.10%) (Figure 2). Further, **AS-1** was entirely ineffective in terms of removing copper(II) ions (%R_ads_ = 0).

After the sorption of heavy metal ions from the aqueous solution on **AS-1**, a desorption process was performed to regenerate the active substrate. The active substrate was immersed in a 0.05 mol/L nitric acid solution, from which the metal ions were washed out. The desorption of the metal ions from the surface of **AS-1** was performed by the procedure described by Witt et al. [29]. It was found that the percentage of desorption of the examined metal ions decreased in the following order: Cr(VI) (69.17%) > Cd(II) (42.35%) > Pb(II) (21.98%) > Ni(II) (39.72%) > Zn(II) (27.45%) > Cu(II) (0.00%).

It is noteworthy that, after desorption in acid and abundant rinsing with water, a regenerated active substrate was obtained without any altered physicochemical properties. This allows a regenerated active substrate that can be reused to reduce the migration of heavy metals to the environment or ground and surface waters to be obtained. The advantages concerning regeneration and the possibility of further reuse applied to all presented solutions. This procedure limits the consumption of raw materials needed to create active substrates while also enabling their reuse, which is consistent with the provisions of the EU Directive [30].

In the second stage of the research, the efficiency of the active substrate was checked in terms of preventing the migration of metal ions to the soil, and then to the aqueous solution located under the soil layer (Experiment 2). This model experiment was conducted to imitate the migration of heavy metal pollutants deep into the soil profile and into groundwater. For this purpose, the active substrate **AS-2** was applied to the soil layer. The solution was passed through both the active substrate and the soil. The concentration of metals was checked after the solution passed through the substrate and then when it passed through the soil. For this reason, it was possible to check to what extent metal ions are eliminated and what percentage of pollutants can enter the soil and then the water. Soil with a grain size of less than 2 mm and a density of 2.2 g/cm^3^ was used for the tests.

The soil contained equal amounts of silt, sand, and clay particles, giving it a loamy texture.

**Figure 3:**
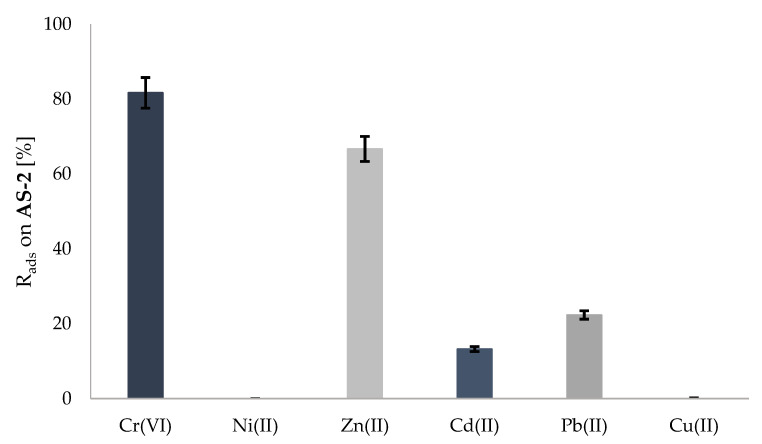
Sorption of metal ions on the active substrate (**AS-2**) according to experiment 2.

After the experiment, which lasted for 24 h (25 °C), it was found that the active substrate showed high efficiency in binding the chromium(VI) and zinc(II) ions at levels of 81.66% and 66.67%, respectively. On the other hand, the %R_ads_ values were much lower in relation to the lead(II) and cadmium(II) ions and amounted to 22.33% and 13.23%, respectively (Figure 3). In this experiment, the copper(II) and nickel(II) ions were not bound by the substrate **AS-2**.

Experiment 2 showed that the active substrate could be successfully used to limit the migration of heavy metal pollutants (especially chromium and zinc) into the soil profile and groundwater.

In the next stage of the study, the active substrate was placed between two layers of soil (Experiments 3 and 4). In experiments 3 and 4, different active substrates (**AS-3** and **AS-4**) were used. The difference between them was related to the applied skeleton material—**AS-3** consisted of fiberglass and **AS-4** of cotton textile.

**Figure 4:**
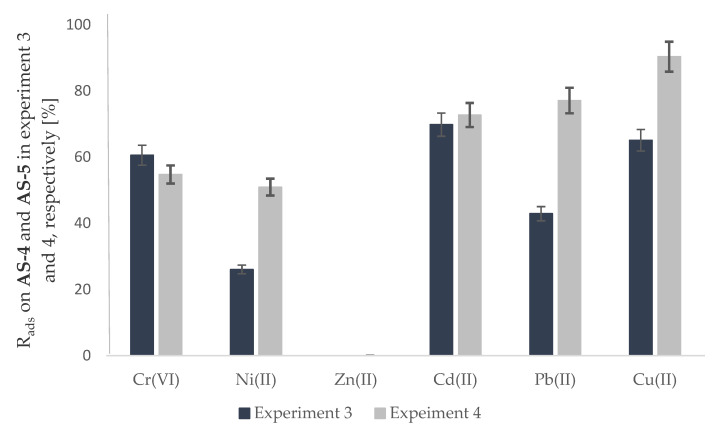
Sorption of metal ions on the active substrate (**AS-4** and **AS-5**) according to Experiments 4 and 5, respectively.

Comparing the obtained test results (Table 2), it is clear that slightly better results were obtained in experiment No. 3, which used the **AS-3** substrate based on fiberglass (Figure 4). Fiberglass is a chemical fiber obtained from water glass, i.e., an aqueous solution of sodium and potassium or sodium and potassium silicates with variable compositions and the general formula of mMe_2_O·nSiO_2_·xH_2_O (where: Me = Na, K). On the other hand, cotton textile, apart from trace amounts of other substances, contains mainly pure cellulose, i.e., an unbranched biopolymer, a polysaccharide built linearly from D-glucose molecules linked by β-1,4-glycosidic bonds. The construction of silane usually makes these compounds more reactive in comparison to the analogous carbon compounds due to the stronger polarization of silicon non-metal bonds compared with carbon non-metal bonds. This may be a possible reason for the better metal ion binding by the active substrate with fiberglass (**AS-3**) than with cotton textile (**AS-4**).

The last stage of the research was related to examining the impact of adding a plasticizer to the active substrates on the metal ion sorption processes. This substance is used in plastic products, reduces the proportion of the crystalline phase to the amorphous phase in the mass of the polymer, and increases its elasticity and flexibility. Therefore, the test results obtained for the unplasticized and plasticized substrates for **AS-4** and **AS-5**, respectively, were compared. More copper(II) (90.39%), lead(II) (77.12%), and nickel(II) ions (50.96%) were adsorbed on the substrate plasticized using bis(2-ethylhexyl)adipate. In turn, the amount of cadmium(II) ions bound by these two substrates was comparable and equaled 69.83% for **AS-4** and 72.76% for **AS-5**, respectively. The amount of chromium(VI) ions adsorbed by **AS-5** was about 5% lower than that adsorbed on **AS-4** and amounted to exactly 54.77%. Figure 4. Shows a comparison of the results obtained for these two substrates.

The addition of an ADO (plasticizer) resulted in the much greater retention of heavy metals on the active substrate. This may have been due to the loosening of the polymer structure and the possibility for better penetration of the active agent Aliquat 336 into its structure. The loosening of the polymer structure probably also increased the active surface area of the substrate, resulting in more of the active sites of Aliquat 336 being ready to bind the metal ions present in the aqueous solution.

### 2.2. Quantification of Aliquat in Soil for Its Leaching from Active Substrates

**Figure 5:**
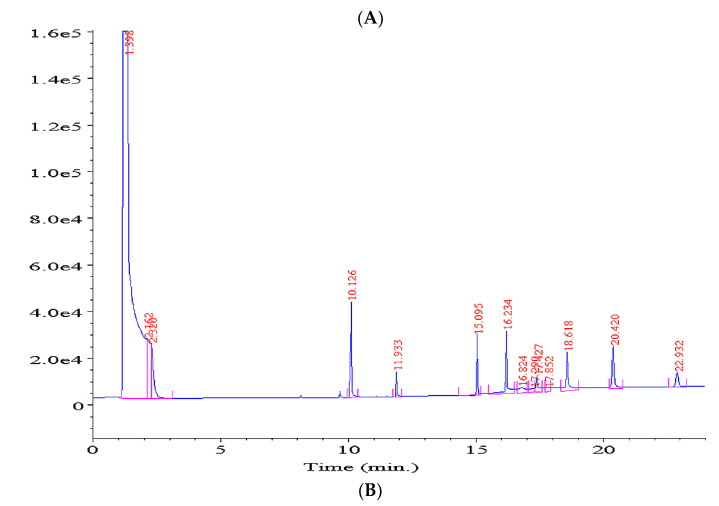

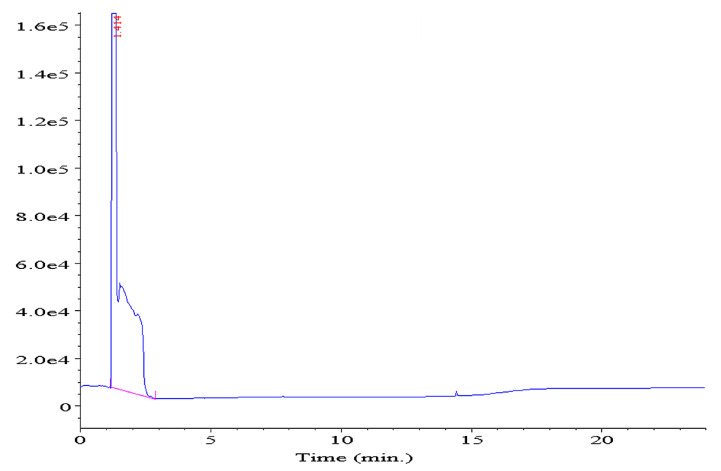
Chromatograms of (**A**) Aliquat 336 with a concentration of 0.1 mg·mL^−1^ and (**B**) the tested soil extract.

In order to make sure that Aliquat 336 is not leached from the active substrate during the sorption process and is safe for the environment, a study was undertaken to analyze Aliquat 336 in the soil after the sorption process. The GC-FID method, characterized by good linearity in the tested concentration range (R^2^ > 0.999), was developed for this purpose. The detection limit was 0.1 μg·mL^−1^, while the quantification limit was 0.3 μg·mL^−1^. The coefficient of variation for the analyte was 3.8%. The recovery for the developed extraction method was 86%. Figure 5A shows a chromatogram of a soil extract into which Aliquat 336 was introduced for method development. The chromatographic analysis of the soil extract after the sorption of heavy metals on the active substrate did not show the characteristic peaks of the substances contained in Aliquat 336 (Figure 5B). Thus, the tested extracts were characterized by lower concentrations of Aliquat 336 than the limits of detection and quantification. Taking into account the fact that the method enabled the determination of trace amounts of Aliquat 336, the absence of peaks in the chromatograms meant the absence of Aliquat 336 in the collected extracts. Based on the analyses carried out, it was found that Aliquat 336 did not penetrate the soil.

## 3. Discussion

The obtained results were compared with the literature data. In relation to the current state of knowledge, it was found that the percentages of sorption of individual metal ions (Cr(VI), Ni(II), Zn(II), Cd(II), Pb(II), and Cu(II)) depended on such factors as the type of metal ion separated from the so-called feed phase solution, the pH of the medium, the type of sorbent and its specific surface, and the contact time of metal ions with the sorbent [31,32,33,34].

Because the investigated active substrates with the addition of Aliquat 336 are an innovative solution used in the soil and soil–water environments, the authors compared the presented results with those achieved using sorbents with the addition of Aliquat 336, which are used to remove heavy metals from solutions. In the literature, Aliquat 336 has previously been used as an additive for polymer inclusion membranes (PIM) and emulsion liquid membranes (ELM), and as an extractant in liquid–liquid extraction. Heavy metals have been removed from aqueous solutions and electrolyte solutions using the above methods, e.g., from batteries [31,32,35,36,37]. Based on the obtained results, it was found that the obtained active substrates enabled a higher recovery of the tested metal ions from multicomponent solutions. Thus, **AS-1** enabled the recovery of approx. 93% of Cr(VI) ions, **AS-2** enabled the recovery of approx. 82% of Cr(VI) ions and approx. 67% of Zn(II) ions, and **AS-5** enabled the recovery of approx. 73% of Cd(II) ions in comparison with the studied literature (Table 3). Differences appeared in the case of Pb(II) ions, where approx. 77% was separated using **AS-5**, even though Kadiv et al. [37] obtained a Pb(II) ion sorption level of 95%. However, it is worth noting that Kadiv et al. [37] carried out the sorption of lead ions from a one-component model solution; as such, there were no competitive reactions in the solution in question. Moreover, Kagaya et al. reported PIM containing Aliquat 336 as an ion carrier for the extraction of Cr(VI) from a solution containing 0.01 mol/L NaNO_3_ at pH 2, which was 92% after 6 h of processes. The investigators found that PIMs could be easily coated onto various materials. The PIM coated on glass beads and packed into a glass tube was applicable to the online preconcentration of thiocyanate [38]. On the other hand, Semghouni et al. designed multi-frame flat sheet membrane contactors (MF-FSMC) consisting of ten parallel frames alternating aqueous and organic phases to be used for chromium(VI) ion removal from aqueous solution. Aliquat-336 was used as a carrier using a polypropylene flat sheet membrane for Cr(VI) removal. Aliquat 336 was the most important parameter, and the extraction efficiency of Cr(VI) was 98.32% [39].

As active substrates can be an alternative to reactive barriers, the results obtained in this research were also compared with the current data on heavy metal removal by PRBs. Yu et al. removed approx. 66% of chromium(VI) ions using a PRB containing a mixture of CTMAB-Z and Fe(0) [40]. In contrast, Khail and Abdalwahedb carried out a sorption process using acetic acid and zeolite–PRB as a purifying solution, removing about 40–57% of nickel(II) ions [41]. Using nanoscale zero-valence iron (NZVI) in PRB as a support material, Liu et al. removed 89.4% of Cr(VI) ions, 98.9% of Cu(II) ions, 94.9% of Cd(II) ions, and 99.4% of Pb(II) ions from wastewater [42]. Generally, using PRB, it is possible to obtain a better sorption efficiency for metal ion removal (over 90%) [33,34] in comparison with active substrates. However, as noted by Zhu et al. [33], the greatest efficiency for PRB was achieved in the case of sorption from one-component solutions. Where two- and four-component solutions were used, the sorption efficiency decreased. Additionally, the effectiveness of PRB depended on the soil and water conditions, e.g., the pH of the matrix and the contact time. The sorption of selected metals may only reach a few percent when the process conditions are not optimal [34]. Usually, the regeneration of PRB and the decomposition of heavy metal salts requires the use of high temperatures, reaching 750 °C to 1000 °C [34]. Nonetheless, the advantages of the presented active substrates over PRBs are the lower dependence on the water and soil conditions, as well as the ease of regeneration and affordable production costs. Moreover, Lee et al. used PRB filled with food waste ash to treat soil contaminated with copper and lead via EK remediation techniques. The removal of copper and lead was most effective after 10 days of operation and 8 days after electrode exchange. The remediation efficiency of copper ions was about 87%, and that in the case of lead was approx. 44% [43]. Additionally, the vegetable fibers of the cabuya in the PRB can have the capacity to retain heavy metals. The removal percentages of heavy metals by cabuya were 90.09% for zinc, 96.60% for cadmium, 99.24% for copper, and 100% for lead [44].

## 4. Materials and Methods

### 4.1. Chemicals

The stock solutions nitrates were prepared using a diluted standard solution. The nitrate standard solutions of Cr(VI), Ni(II), Cu(II), Zn(II), Cd(II), or Pb(II) with a concentration of 1000 mg/L were of analytical grade and were purchased from Sigma Aldrich (St. Louis, MO, USA). The poly(vinyl chloride) (PVC) in suspension with an average molecular weight of 72,000 was obtained from Anwil (Włocławek, Poland). Aliquat 336 (methyltrioctylammonium chloride) was purchased from Thermo Scientific (Waltham, MA, USA). The bis(2-ethylhexyl)adipate (ADO), tetrahydrofuran, nitric acid, hydrochloric acid, and methanol (all of analytical grade) were sourced from Avantor (Gliwice, Poland) and were used without further purification. Fiberglass (Achitex Minerva, Poland) and cotton textile (Matopat, Poland) were obtained from local Polish producers.

### 4.2. Procedure for Cleaning and Preparing the Soil for Research

The soil sample taken contained 2.5% organic carbon. The organic matter content was tested according to PN-ISO 14235: 2003. The soil was ground in a mortar and sieved through a 2 mm sieve to separate the structural parts (gravel and stones) from the earth parts. During the test, which was conducted to check whether Aliquat 336 migrates from the active substrates to the soil, extraction was also performed to clean the soil of organic pollutants. The screened fraction was extracted 3 times using 10% hydrochloric acid, then washed with water to pH 7. The next step was a 2-fold extraction with methanol. The cleaned soil was dried in a laboratory drier at 40 °C.

### 4.3. Preparation of Active Substrates (ASs)

The active substrates (ASs) were prepared in three different ways:

Preparation of active substrates **AS-1**, **AS-2**, and **AS-3**

To prepare **AS-1**, **AS-2**, and **AS-3**, a solution containing 65 wt.% of poly(vinyl chloride) as a matrix and 35 wt.% of Aliquat 336 as an ion carrier was prepared in 10 mL of tetrahydrofuran. The fiberglass in the form of a strip with small, square holes was dipped into the obtained mixture. Then, the excess mixture was removed from the fiberglass tape. **AS-1**, **AS-2**, and **AS-3** were formed after 24 h with all of the tetrahydrofuran having evaporated by that time. The solvent was then evaporated in an inert gas (nitrogen) stream using a Lipopharm concentrator. The mass of the obtained **AS-1**, **AS-2**, and **AS-3** was 0.350 g.

Preparation of active substrate **AS-4**

A solution of 74 wt.% poly(vinyl chloride) as support and 26 wt.% Aliquat 336 was prepared in 10 mL of tetrahydrofuran and a piece of cotton textile was dipped into it. The excess mixture was then removed from the textile. **AS-4** was formed after 24 h with all of the tetrahydrofuran having evaporated by that time. The mass of the obtained **AS-4** was 0.918 g.

Preparation of **AS-5**

To prepare **AS-5**, a solution of 58 wt.% poly(vinyl chloride) as a support, 22 wt.% Aliquat 336, and 20 wt.% bis(2-ethylhexyl)adipate as a plasticizer was prepared in 10 mL of tetrahydrofuran. A piece of cotton textile was then dipped in the obtained solution, with any excess mixture subsequently removed from the textile. **AS-5** was formed after 24 h with all of the tetrahydrofuran having evaporated by that time. The mass of the obtained **AS-5** was 1.082 g.

### 4.4. Preparation of Aqueous Solutions

The aqueous phases were prepared from standard solutions of six different metal ions with concentrations of 1000 mg/L:

Solution 1 (**S-1**)

Monometallic solutions of nitrates with each metal ion (Cr(VI), Ni(II), Cu(II), Zn(II), Cd(II), or Pb(II)) with a concentration of 10 mg/L were prepared using appropriate amounts of a standard solution with a concentration of 1000 mg/L diluted with water.

Solution 2 (**S-2**)

Polymetallic solutions of metal ion nitrates (Cr(VI)–Ni(II)–Cu(II)–Zn(II)–Cd(II)–Pb(II)) with concentrations of 10 mg/L were prepared by adding the appropriate amounts of the standard solutions of each metal ion with concentrations of 1000 mg/L and diluting them with water.

Solution 3 (**S-3**)

Polymetallic solutions of metal ion nitrates (Cr(VI)–Ni(II)–Cu(II)–Zn(II)–Cd(II)–Pb(II)) with concentrations of 5 mg/L were prepared by adding the appropriate amounts of standard solutions of each metal ion with concentrations of 1000 mg/L and diluting them with water.

### 4.5. Methodology of the Sorption of Metal Ions onto Active Substrates

Experiment 1

**Figure 6:**
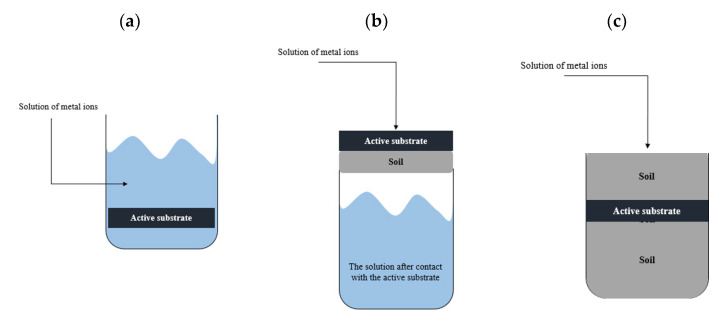
Graphical presentation of the sorption processes: (**a**) Experiment 1, (**b**) Experiment 2, and (**c**) Experiments 3, 4, and 5.

The first experiment aimed only to check whether the synthesized substrate can bind metal ions at all. Therefore, **AS-1** with a mass of 0.350 g was immersed in 50 mL of solution **S-2** (Figure 6a). After 24 h, **AS-1** was removed from the solution and its metal ion content was determined.

Experiment 2

A total of 30 mL of the one-component metal ion solution **S-1** (Figure 6b) was poured over 10 g of the soil, which was placed under **AS-2** so a maximum of 0.3 mg of each metal could pass into the 10 g of the soil.

Experiment 3

Active substrate **AS-3** was placed between two soil layers (Figure 6c). The soil’s top layer weighed 80.016 g, whereas the bottom layer weighed 100.267 g. A total of 100 mL of the **S-3** metal ion solution was poured into the prepared system, i.e., a maximum of 0.5 mg of a given metal could pass into the soil.

Experiment 4

Active substrate **AS-4** was placed between two soil layers (Figure 6c). The soil’s top layer weighed 36.159 g and the bottom layer weighed 99.7 g. A total of 100 mL of the **S-3** metal ion solution was poured into the system; therefore, the soil sample could react with up to 0.5 mg of a given metal.

Experiment 5

Active substrate **AS-5** was placed between the soil layers (Figure 6c). The soil’s top layer weighed 35.362 g and the bottom layer weighed 100.851 g. Then, 100 mL of the **S-3** metal ion solution was poured into the system; thus, the soil sample could react with up to 0.5 mg of a given metal.

The soil from experiments 2–5 was then analyzed for the presence of metals. In order to do this, it had to be mineralized. This was achieved using 28 mL of aqua regia per 3 g of soil, with the filtrate being transferred to a 50 mL volumetric flask each time. The metal content of this volume was determined using the AAS method (atomic absorption spectrometry). The uncontaminated soil sample was also analyzed.

### 4.6. Quantification of Soil Using Gas Chromatography to Determine the Elution of Aliquat 336 from the Active Substrates

Aliquat 336 is a quaternary amine salt that contains a mixture of C8 (octyl) and C10 (decyl) hydrocarbon chains with a predominance of C8.

In order to perform the quantitative analysis of Aliquat 336, a calibration curve was determined in the form of a linear function y = ax + b, where y represents the sum of the area under the peak of all compounds included in the sample, while x is the concentration of the analyzed substance. The calibration curve was determined over a concentration range of 0.1 µg/L to 2.0 mg/L. The concentration range considered during the determination of the calibration curve corresponded to the concentration ranges of methyltrioctylammonium chloride used in the prepared active substrates. Aliquat 336 was determined using an Agilent 5977A gas chromatograph with a flame ionization detector (FID). An HP-5MS column (0.25 mm × 30 m × 0.25 μm) was used for the tests. The analyses were performed under the following chromatographic conditions: injection port temperature of 250 °C, detector temperature of 280 °C, and oven temperature program from 50 °C/4 min, where it increased from 15 °C/min to 30 °C (maintained for 4 min). Helium was used as the carrier gas and the gas flow was set at 1 mL/min. The volume of the dosed sample was 1 μL. To verify that Aliquat 336 did not migrate to the soil, the soil sample was extracted using 50 mL of methanol and filtered; the extract obtained was then concentrated to 2 mL under a stream of nitrogen using a Lipopharm concentrator. Finally, the extract was chromatographically analyzed.

### 4.7. Determination of Metals in the Water Solution and Soil Filtrate by the AAS Method

Atomic absorption measurements were carried out using a Thermo Scientific ICE 3000 atomic absorption spectrometer. Deuterium background correction was used; the radiation source consisted of hollow cathode single-element lamps with a current of 4.0 mA. An air/acetylene mixture was used for the determinations. The determinations were performed in the flame mode at the following wavelengths: 357.9 nm for chromium, 232.0 nm for nickel, 324.8 nm for copper, 213.9 nm for zinc, 228.8 nm for cadmium, and 217.0 nm for lead. Commercially available one-element metal standard solutions were used for calibration in the concentration range of 0.1–5.0 mg·L**^−^**^1^. The correlation coefficients for all calibration curves were not lower than 0.995.

## 5. Conclusions

This paper presents solutions (**AS**s) that effectively reduce the concentration of heavy metals in water as well as in the soil and water-soil environments under model conditions. It was found that, in the aqueous solution, the active substrate **AS-1** decreased the concentrations of nickel, cadmium, and lead by more than half; in the case of chromium, this reduction exceeded 90%. High efficiency in reducing metal migration from soil to water (with **AS-2** applied) was also noted, especially for chromium and zinc (reduction exceeding 81% and 66%, respectively). In the soil environment, active substrate **AS-5** with the addition of plasticizer showed the greatest effectiveness. This solution resulted in a reduction of each tested metal ion by at least 50%, and of cadmium, lead, and copper by over 70%.

Apart from the demonstrated effectiveness in reducing the concentration of metals in environmental matrices, the production of the **AS** active substrates is economical, and they can be regenerated and reused in further sorption cycles. The indicated advantages make the active substrates (**AS-1**–**AS-5**) an alternative to the solutions currently available on the market.

## 6. Patents

This manuscript is based on Polish patent application no. P.442582.

## Figures and Tables

**Table 1:** Sorption capacities (q_t_, mg/g) of the investigated active substrates (ASs) depending on metal ion binding. The concentration of metal ions for **S-1** and **S-2** was 10 mg/L, and that for **S-3** was 5 mg/L. For experiments with **AS-1**, **AS-2**, **AS-3** and **AS-5**, and **AS-4**, the **S-2**, **S-1**, **S-3**, and **S-4** solutions were used, respectively. The details are described in paragraph 4.3 and paragraph 4.4.

Metal Ions	q_t_, mg/g				
	AS-1	AS-2	AS-3	AS-4	AS-5
Cr(VI)	1.34	0.70	1.81	1.32	1.01
Ni(II)	0.78	0.00	0.83	0.57	0.94
Zn(II)	0.40	0.57	0.00	0.00	0.00
Cd(II)	0.81	0.11	1.72	1.51	1.34
Pb(II)	0.82	0.19	1.31	0.94	1.43
Cu(II)	0.00	0.00	0.00	0.71	0.84

The tolerance of the given values of q_t_, is ±0.01.

**Table 2:** Comparison of the results of %R_ads_ obtained in Experiments 3 and 4.

Experiment No./Active Substrates	% R_ads_, %					
	Cr(VI)	Ni(II)	Cu(II)	Zn(II)	Cd(II)	Pb(II)
3/**AS-3**	63.22	37.77	0	0	78.01	45.91
4/**AS-4**	60.59	26.06	65.11	0	69.83	42.92

The tolerance of the given values of % R_ads_ is ±0.01.

**Table 3:** Comparison of the sorption efficiencies of heavy metals on **AS-1**–**AS-5** with the literature data [%] [30,31,32,33,34,35,36].

%R_ads_, %									
Metal Ions	AS-1	AS-2	AS-3	AS-4	AS-5	PRB	PIM	ELM	SE
Cr(VI)	93.50	81.66	63.22	60.59	54.77	95.65	77.34	-	-
Ni(II)	54.30	0	37.77	26.06	50.96	5–80	-	1.50	92.20
Zn(II)	28.10	66.67	0	0	0	5–98	-	28.00	-
Cd(II)	56.70	13.23	78.01	69.38	72.76	-	11–90	90.50	-
Pb(II)	57.10	22.33	45.91	42.92	77.12	-	-	95	-
Cu(II)	0	0	0	65.11	0	92–99	-	-	71.60

The tolerance of the given values of % R_ads_ is ±0.01.

## Data Availability

The data presented in this study are available on request from the corresponding author.

## Sample Availability

Samples of the compounds are available from the authors.
